# Pulmonary sarcoidosis with arterial involvement

**DOI:** 10.4322/acr.2021.294

**Published:** 2021-07-08

**Authors:** Adriane Souza da Paz, João Carlos Coelho, Bartira Melo, Ana Luísa Pedreira, Mittermayer Barreto Santiago

**Affiliations:** 1 Hospital Universitário Professor Edgard Santos, Serviço de Reumatologia, Salvador, BA, Brasil; 2 Fundação José Silveira, Serviço de Patologia, Salvador, BA, Brasil; 3 Hospital Santa Izabel, Serviço de Reumatologia, Salvador, BA, Brasil

**Keywords:** Granuloma, Granulomatosis with Polyangiitis, Sarcoidosis

## Abstract

Necrotizing sarcoid granulomatosis (NSG) is a rare and under-recognized cause of granulomatous disease, described as a variant of typical nodular sarcoidosis. It can be asymptomatic when the patient has a single pulmonary nodule or may be accompanied by cough, fever, and dyspnea, or even symptoms due to the involvement of other organs such as the eyes, liver, and central nervous system. The histopathological analysis is essential for the differential diagnosis of other infectious and non-infectious causes of granuloma and to determine the appropriate treatment. NSG is characterized by the presence of a granuloma with extensive coagulative necrosis associated with the occurrence of vasculitis. We present the case of a patient diagnosed with NSG who had an unusual outcome with recurrent pulmonary thromboembolisms followed by hemodynamic instability and death.

## INTRODUCTION

Necrotizing sarcoid granulomatosis (NSG) is a rare entity of unclear etiology. NSG was first described by Liebow,^1^ in 1973, as a granulomatous disease with features of sarcoidosis and Wegener's granulomatosis (currently known as granulomatosis with polyangiitis [GPA]) in a patient with a single mass in the lung. Some authors consider NSG to be a variant of sarcoidosis, with different epidemiological, clinical, and pathological features. Above all, NSG shows a histopathological pattern of more severe necrosis and vasculitis.^2^ However, other authors suggest that necrosis represents a final stage of the typical sarcoidosis when the vascular obstruction leads to necrosis.^3^

NSG is a systemic disease, involving predominantly the lungs. NGS is generally presented as an asymptomatic mass or with clinical features such as fever, cough, dyspnea, and chest pain.^3^ It may also involve the eyes, liver, spleen, and central nervous system. The diagnosis is commonly based on the histopathology, and despite the multisystemic involvement, NSG has a benign clinical course with a good response to corticoid therapy.^2^

Herein, we describe a patient with NSG whose diagnosis was based on the exploration of a pulmonary nodule identified by chest imaging. This is the second case reported in Brazil.

## CASE REPORT

**Figure 1:**
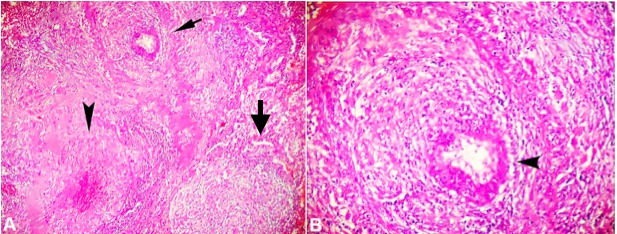
Photomicrographs of the pulmonary nodule. **A –** Shows a granuloma (right arrow), a granuloma with fibrinoid necrosis (arrowhead) and a granulomatous angiitis (left arrow); **B –** Shows granulomatous angiitis in higher magnification (black arrowhead). Fungus and mycobacteria were not found (both pictures H&E, A 100X, and B 200X).

A 43-year-old woman reported progressive dyspnea and occasional dry cough for the last 2 years. Her mother had been previously diagnosed with “pulmonary sarcoidosis”. Six months before her first hospital admission, she experienced episodes of severe pain in the posterior region of the right hemithorax. During the outpatient investigation, a cardiac cause for her discomfort was excluded. Chest radiography revealed hypotransparency in the lower 2/3 of both hemithoraces. The chest computed tomography revealed a nodule with solid and non-calcified density, lobulated contours, measuring 2.7 × 1.4 cm, located in the right upper lobe in close contact with the horizontal fissure, aligned with the parietal pleura. Two other 0.7-cm non-specific nodules were identified. During hospitalization, physical examination showed that the patient was lucid and without neurological changes. No palpable lymph nodes or skin lesions were observed. A respiratory rate of 26 breaths per minute, heart rate of 100 beats per minute, and blood pressure of 110/70 mmHg were recorded. A vesicular murmur was present in both lungs and pulmonary rales in the left lung base. There were no changes in the abdomen or the musculoskeletal system. Laboratory tests revealed a hemoglobin of 13 g/dL (reference range [RR]; 12-16mg/dl); white blood cell count of 10.630 cells/mm^3^ (RR; 4.000-12.000) with 60% neutrophils and 310.000/mm^3^ platelets (RR; 150.000-450.000); C-reactive protein of 192 (RR;3-5 mg/dL); and erythrocyte sedimentation rate of 32 mm/h (RR; <20mm/h). There were no changes in renal function, hepatic enzymes, or hydro electrolytic disorders. Immunological and serological tests were negative, including antinuclear antibodies, anti-SSA, anti-SSB, anti-DNA, anti-Sm, rheumatoid factor, and ANCA. The lingula of the lung was biopsied using video thoracoscopy. The patient developed a persistent air leak in the chest drain, and 14 days after the elective procedure, she underwent a new surgical approach. Histopathological analysis revealed “angiocentric granulomatosis compatible with Wegener's granulomatosis or necrotizing sarcoid granulomatosis” in the first specimen and NSG in the second sample. The Grocott, PAS, and ZN staining ruled out eventual infection by fungus or mycobacterium (Figure 1).

**Figure 2:**
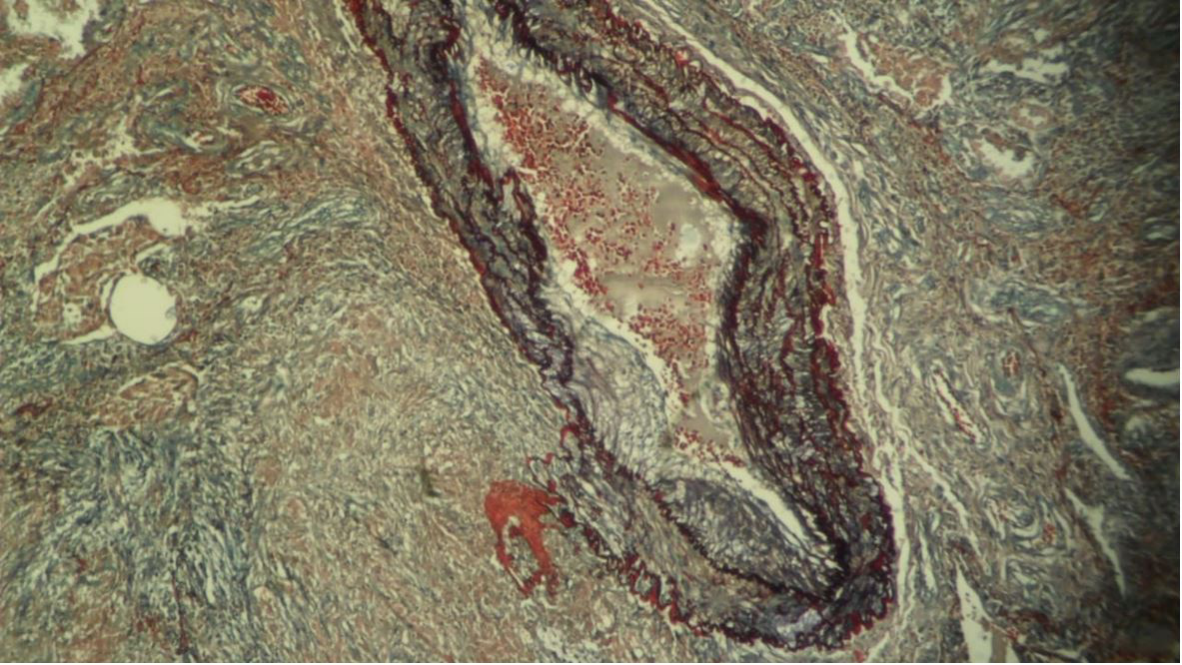
Photomicrograph of the pulmonary nodule showing a vessel injury and granuloma (Weigert-Van Gieson, 100X).

To confirm the destruction of the vessel wall and the presence of granuloma, we also performed Weigert-Van Gieson staining (Figure 2).

Subsequently, 1 mg/Kg prednisone was administered. However, dyspnea worsened, and a new investigation revealed massive thromboembolism with hemodynamic and cardiac repercussions, and despite the adopted therapeutic measures, the patient died. The patient had no risk factors for thrombosis, except the surgical procedure, and, unfortunately, we were not able to search for acquired or hereditary causes of thrombophilia.

## DISCUSSION

NSG is an under-recognized condition, characterized by a necrotizing granuloma with vasculitis.^2^ NGS is not limited to a specific age group; however, it is more frequently observed among women at the age of 40 years. Its pathogenesis remains unclear, but it is suspected that the condition results from the association of genetic background and environmental triggers.^4^ Karpathiou et al.,^3^ identified 130 cases described in the literature.^3^ Based on their study, we believe that this is the second such case described in Brazil. The first case, reported by Lazzarini et al.,^5^ referred to a 15-year-old patient with pleuritic pain, dry cough, hemoptysis, and dyspnea, and the investigation identified pulmonary nodules with cavitation. Three members of the patient’s family had the diagnosis of sarcoidosis previously.^5^ Positive family history appears to be a crucial predisposing marker for sarcoidosis and its variants,^3^ as it was also the case in the present patient.

Pulmonary granuloma is the main clinical presentation of the disease, as illustrated in this case report. It can appear as an asymptomatic mass, in about 25% of the cases, or accompanied by respiratory symptoms, such as cough, dyspnea, and chest pain.^6^ There may also be ocular, hepatic, splenic, and central nervous system involvement.^2^^,^^6^^,^^7^

The differential histopathological diagnoses include tuberculosis and fungal infections such as histoplasmosis, coccidioidomycosis, and aspergillosis. These were ruled out in the present case by the histological examination and by serological exams, and the treatment would be radically different. Furthermore, we should also exclude diseases in which granuloma is a common finding, such as GPA, where vasculitis may be found in sparse foci and at a distant location from the necrotizing masses. Conversely, in NSG, vasculitis is found in the foci of necrosis in large nodular masses.^8^ Our patient had a negative ANCA test, besides the typical histopathology of NSG. The greatest challenge in the differential diagnosis is nodular sarcoidosis (typical) since both conditions may have nodular aggregates of epithelioid cell granulomas and granulomatous vasculitis. However, from a practical point of view, such differentiation is of limited relevance as both conditions respond similarly to the therapeutic regimens.

Generally, NSG has a benign clinical course, and the patients show a positive response to corticosteroid therapy. While some cases may require surgical excision, others may resolve spontaneously. In the present case, the patient’s outcome was atypical with the development of severe thromboembolic complications, which we attributed to the surgical intervention since there is no evidence in the literature that NSG predisposes to such clinical outcomes.

In conclusion, NSG is a rare condition and may resemble pulmonary neoplasia when a pulmonary mass is observed by imaging examination or other granulomatous diseases by histopathological examination. This differentiation is crucial for avoiding inadequate treatment.

## References

[1] Liebow AA (1973). The J. Burns Amberson lecture--pulmonary angiitis and granulomatosis. Am Rev Respir Dis.

[2] Parejo-Morón AI, Tornero-Divieso ML, Fernandez-Diaz MR, Munoz-Medina L, Preda O, Ortego-Centeno N (2020). Necrotizing sarcoid granulomatosis: a disease not to be forgotten. Case Rep Med.

[3] Karpathiou G, Batistatou A, Boglou P, Stefanou D, Froudarakis ME (2018). Necrotizing sarcoid granulomatosis: A distinctive form of pulmonary granulomatous disease. Clin Respir J.

[4] Chittock DR, Joseph MG, Paterson NA, McFadden RG (1994). Necrotizing sarcoid granulomatosis with pleural involvement. Clinical and radiographic features. Chest.

[5] Lazzarini LC, FATeixeira MFA, Rodrigues RS, Valiante PMN (2008). Necrotizing sarcoid granulomatosis in a family of patients with sarcoidosis reinforces the association between both entities. Respiration.

[6] Shibata T, Takahashi K, Uchida M, Yamasaki F, Kawashima M, Sueoka-Aragane N (2018). Necrotizing sarcoid granulomatosis with natural resolution after a surgical lung biopsy. Intern Med.

[7] Popper HH, Klemen H, Colby TV, Churg A (2003). Necrotizing sarcoid granulomatosis--is it different from nodular sarcoidosis?. Pneumologie.

[8] Churg A, Carrington CB, Gupta R (1979). Necrotizing sarcoid granulomatosis. Chest.

